# Treatment of cervical cancer in HIV-seropositive women from developing countries: a protocol for a systematic review

**DOI:** 10.1186/s13643-018-0686-9

**Published:** 2018-01-25

**Authors:** Witness Mapanga, Tsungai Chipato, Shingairai A. Feresu

**Affiliations:** 1 School of Health Systems and Public Health, Epidemiology & Biostatistics, University of Pretoria, 5-10 H.W. Snyman Building, Pretoria, South Africa; 2 College of Health Sciences, University of Zimbabwe-University of California, San Francisco Collaborative Research Programme, University of Zimbabwe, Avondale, Harare, Zimbabwe; 3 47 Newstead Road, Harare, Marlborough Zimbabwe

**Keywords:** Developing countries, Cervical cancer, HIV, Treatment

## Abstract

**Background:**

Cervical cancer has become the most common cancer affecting women in Africa. Significantly, 85% of these annual deaths occur in the developing world, with the majority being middle-aged women. Research has shown that in sub-Saharan Africa, cervical cancer trends are on the rise in the past two decades because of HIV and this has resulted in an increase in cervical cancer cases among young women. However, little or no information exists that has shown that any of the available treatment methods are more effective than others when it comes to treating cervical cancer in HIV-seropositive women. The aim of this protocol is to offer a plan on how to systematically review cervical cancer treatment methods available for HIV-seropositive women in developing countries.

**Methods/design:**

The Preferred Reporting Items for Systematic Reviews and Meta-Analyses Protocols (PRISMA-P) statement was used to develop the protocol for the systematic review which will be reported in accordance with the PRISMA guidelines. A number of databases, Embase, MEDLINE, PubMed, CINAHL and Cochrane Library, will be searched for relevant studies, and citation and reference list tracking will be used to search for additional studies. Prospective and retrospective cohort studies, case-control, randomised controlled trials and cross-sectional studies that were carried out in and for the developing world will be eligible for inclusion. Peer-reviewed studies and grey literature examining cervical cancer treatment modalities in HIV-seropositive women will be included. Descriptive statistics and tables will be used to summarise results, and meta-analysis will be used where appropriate.

**Discussion:**

The review findings will provide the current picture of the existing treatment methods being used to treat cervical cancer in HIV-seropositive women in developing countries. The findings might be used for the establishment of evidence-based guidelines for treatment of cervical cancer in seropositive women as well as prompt policy-makers and governments to decide and support future research in a way to find a lasting solution.

**Systematic review registration:**

PROSPERO CRD42017054676

https://www.crd.york.ac.uk/PROSPERO/display_record.php?RecordID=54676

**Electronic supplementary material:**

The online version of this article (10.1186/s13643-018-0686-9) contains supplementary material, which is available to authorized users.

## Background

Progress in the prevention and treatment of cervical cancer has been made but challenges still exist in developing countries [1, 2]. Low-resource settings are faced with challenges of financial resources, poor and none existent health infrastructure (laboratories, cervical cancer screening centres), lack of technology and few qualified health personnel mainly due to the brain drain [1]. These challenges coupled with lack of proper epidemiological data, lack of knowledge and inadequate information on cervical cancer in low-resource settings have created a major public health issue that is threatening to derail the progress made under the millennium development goal (MDG) number five; reducing maternal mortality and achieving universal access to reproductive health.

The adverse effects of HIV in most low-resource settings have increased the burden of cervical cancer [2–5]. Like most opportunistic infections, HIV-seropositive women are at higher risk of HPV infection due to their immune compromised status with a risk 2 to 12 times more when compared to HIV-negative women [3–5].

Since current treatment modalities for precancerous lesions and cervical cancer are based on the stage of the lesion and available resources, the associated poor outcomes of treatment among HIV-seropositive women in developing countries may be due to a lack of optimal treatment regimen [6]. Most developing countries lack skilled surgeons to carry out radical surgery for cervical cancer and this has left HIV-seropositive women with cervical cancer with few treatment options. In cases where surgeons are available, surgery is expensive and out of reach of many, who happen to be poor [7]. In developing countries especially sub-Saharan Africa, many women with cervical cancer have no access to radiotherapy, further limiting their treatment options.

However, little or no information exists that has shown that any of the treatments are more effective than others when it comes to treating cervical cancer in HIV-seropositive women. In sub-Saharan Africa, treatments like radiation therapy and other surgical procedures are not fully utilised because of lack of equipment and qualified personnel, hence little has been documented on which treatment procedures are being used for cervical cancer in HIV-seropositive women [8]. There is lack of evidence-based guidelines and strategies for screening, vaccination against HPV, prevention and treatment of cervical cancer in HIV-seropositive women in most developed countries [9, 10]. Coupled with this, there is little rigorous evidence on the global epidemiology of the treatment of cervical cancer in HIV-seropositive women [8]. Therefore, we aimed to review the different treatment methods being used to treat cervical cancer in HIV-seropositive women in developing countries.

## Methods/design

The PRISMA-P statement (see attached PRISMA-P statement) guided the development and reporting of this protocol [11] whilst the systematic review will be reported according to the Preferred Reporting Items for Systematic Reviews and Meta-Analyses (PRISMA) guidelines [12].

### Protocol registration

This review protocol is registered in PROSPERO database (registration number: CRD42017054676, [13], https://www.crd.york.ac.uk/PROSPERO/display_record.php?RecordID=54676).

### Studies’ eligibility criteria

Studies will be included if

- Cervical cancer treatment methods for HIV-positive women (such as chemotherapy, radiation therapy, surgery, cryotherapy and targeted therapy among others)
- Cervical cancer treatment methods and HIV are considered being independent and outcome variables
- Published in peer-reviewed journals and grey literature (conferences, dissertations, government health reports)
- They were done in or for countries or regions that are considered developing by the United Nations [14]
- They are observational study designs (retrospective cohorts, prospective cohorts, cross-sectional and case-control) or randomised controlled trials
- Studies done across developed and developing countries, the team will follow the guidance provided by Mapanga and colleagues [15]. The review team will extract results from the developing countries where it is possible and will contact study authors for more information if it is not available.

Studies will be excluded if they are describing cervical cancer in general, their samples are unrepresentative (non-parametric tests as alluded to in the previous protocol, will be used to determine unrepresentative samples) or if they are reviews [15]. No studies will be excluded because of the length of the follow-up period; instead, follow-up periods will be used to assess the quality of the study outcomes [15]. Non-English language studies, reports and dissertations will also be sought as part of the search strategy and translation of data will be performed by a volunteer where feasible.

### Search strategy

**Fig. 1:**
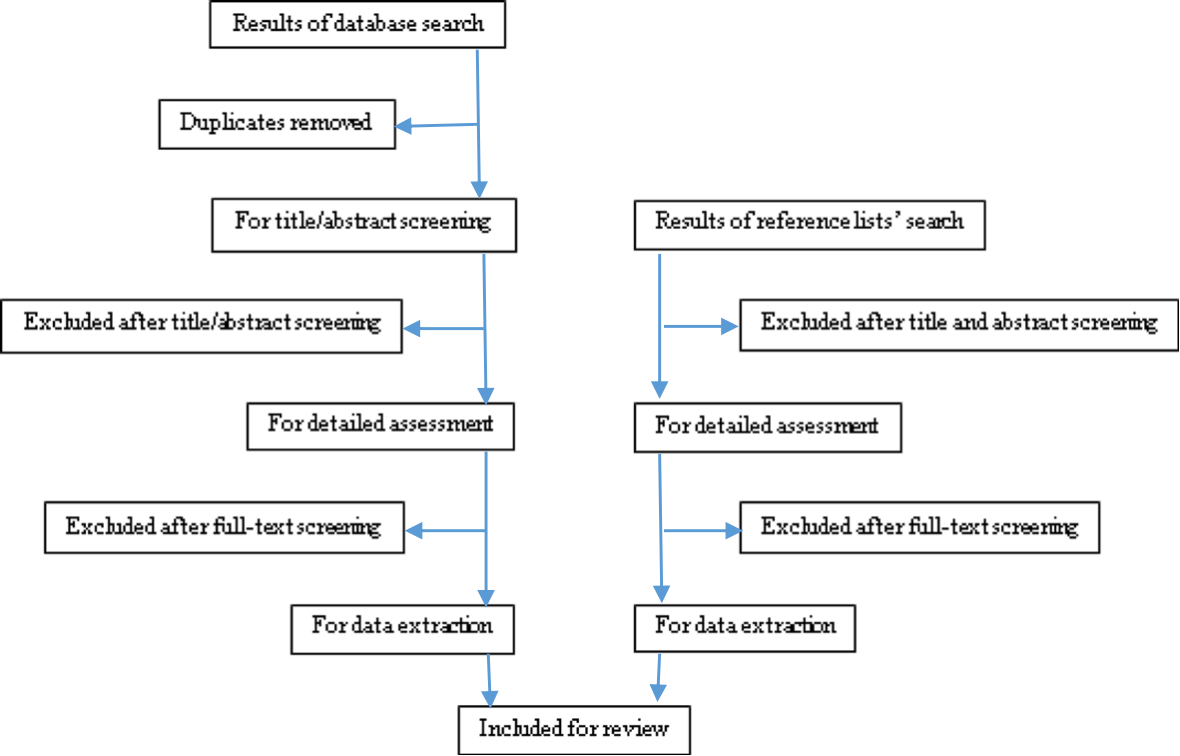
PRISMA review flowchart

The search strategy of the online databases will be based on the criteria developed in the previous review protocol by Mapanga and colleagues [15]. MEDLINE (1966–present) and Embase (1980–present) will be searched via the OVID interface as indicated in Table 1. In addition, PubMed, Cochrane and CINAHL (1961–present) will be searched using a combination of the following keywords: cervical cancer, treatment, developing countries (the geographical search concept will be extended to include country names of developing countries), invasive cervical cancer, HIV, management of cervical cancer, chemotherapy, surgery and radiation. In addition, health databases which cover developing countries (3ie Systematic Reviews, WHO library and databases, World Bank website) and databases and websites containing on-going research (such as WHO ICTRP and cliniccaltrials.gov) will also be searched for relevant literature. Proximity operators, Boolean logic operators and truncation commands (see Table 2) will be used as suggested in the previous review protocol as well as conducting preliminary search trials [15]. To search for additional and relevant papers, reference and citation tracking will be conducted as indicated in the PRISMA flow diagram (Fig. 1).

**Table 1:** MEDLINE and Embase search strategy

Search Terms	
1. Cervi* canc*.mp. [mp = title, abstract, full text, caption text] 2. cervi* neoplas*.mp. [mp = title, abstract, full text, caption text] 3. cervi* carcinom*.mp. [mp = title, abstract, full text, caption text] 4. cervi* dysplas*.mp. [mp = title, abstract, full text, caption text] 1. 5.cervi* intraepithelial neoplas*.mp. [mp = title, abstract, full text, caption text] 5. treat* or therap*.mp. [mp = title, abstract, full text, caption text] 6. chemotherap* .mp. [mp = title, abstract, full text, caption text] 7. surger*.mp. [mp = title, abstract, full text, caption text] 8. radiation adj3 therap*.mp. [mp = title, abstract, full text, caption text] 9. cryotherap*.mp. [mp = title, abstract, full text, caption text] 10. HIV positive.mp. [mp = title, abstract, full text, caption text] 11. hiv seropositiv*.mp. [mp = title, abstract, full text, caption text] 12. hiv.mp. [mp = title, abstract, full text, caption text] 13. developing countr*.mp. [mp = title, abstract, full text, caption text] 14. underdeveloped countr*.mp. [mp = title, abstract, full text, caption text] 15. low income countr*.mp. [mp = title, abstract, full text, caption text] 16. low resource countr*.mp. [mp = title, abstract, full text, caption text] 17. low resource setting*.mp. [mp = title, abstract, full text, caption text] 18. developing countries.mp. [mp = title, abstract, full text, caption text] 19. 1 or 2 or 3 or 4 or 5 20. 6 or 7 or 8 or 9 or 10 21. 11 or 12 or 13 22. 14 or 15 or 16 or 17 or 18 or 19 2. 24.20 and 21 and 22 and 23

**Table 2:** Techniques to be used in the online databases search

Techniques	Description	Example
Free-text synonyms of keyword search	All known synonyms of the keyword in both British and US spellings	Cervical cancer synonyms: cervical carcinomas, cervix neoplasms, cervical intraepithelial neoplasia, cervix dysplasia etc.
Truncation commands	Using the root word to capture alternative word endings	Cervi* carcinom* searches for words such as cervical carcinoma, cervix carcinomas etc.
Proximity operators	Operators used will be Adj3 in OvidSP interface	hpv adj3 vaccin*
Boolean logic operators	‘OR’ and ‘AND’ will be the two commands to be used. ‘OR’ is used to locate articles with at least one of the search terms. ‘AND’ is to be used near the end of a search so as to combine results of different search concepts.	treat* or therap* OR radiation adj3 therap*. (treat* or therap* OR radiation adj3 therap*) AND (HIV positive OR hiv seropositiv* OR hiv) AND (developing countr* OR underdeveloped countr*)

### Study selection

Two independently working reviewers (from among WM, TC and SF) will merge the results of the electronic database, citation and reference searches as well as screen for abstracts. An additional full-text screening form (see Additional file 1) is going to be used to identify and select potential papers for the review. The two reviewers will make sure the papers are double screened and document reasons that they have for exclusion [15]. The three reviewers (WM, TC and SF) will solve all disagreements and other issues around the screening process through discussions.

### Data extraction

The data extraction process and choice of indicators to be extracted from the included studies will be guided by the process indicated in the previous review [15]. There will be double data extraction by WM, TC and SF and the team will solve disagreements and discrepancies through discussions. An additional data extraction form (see Additional file 2) which will be used to extract data from the identified studies will be pretested and adjusted accordingly. The team will extract the following data from included studies: title of the study, study setting, publication year, study design, exposures, sample size, outcomes, descriptive statistics, risk/odds ratios, and confounders, results of linear and logistic regression.

### Quality assessment

The assessment of the quality of included studies will be guided by a modified version of the Newcastle-Ottawa Quality Assessment Scale (see Additional file 3) [16]. Quality of studies will be assessed according to the criteria suggested by Mapanga and colleagues [15]. All included studies will be assessed based on their study design that was used to measure cervical cancer treatment, validity of key findings (if study is describing cervical cancer treatment methods or a comparison of treatment methods), follow-up period and sample representativeness.

Randomised controlled trials will be assessed according to the criteria in Table 3, whilst observational studies with a control group will be assessed according to Table 4, observational studies without control groups will be assessed according to Table 5 and the quality of the studies’ outcomes will be assessed according to Table 6.

**Table 3:** Randomised clinical trials quality assessment checklist

Assessment criteria	Studies fulfilling criteria	Studies not fulling criteria
Randomisation of participants is reported		
All participants who entered the study would have been accounted for in the analysis		
Participants were analysed in the groups they were randomised to		
Blinded outcome assessment was used		
Power calculation information was provided		
Baseline characteristics of study groups were balanced or adjustment for the imbalance in analyses

**Table 4:** Observational studies with a control group quality assessment checklist

Assessment criteria	Studies fulfilling criteria	Studies not fulling criteria	Studies not applicable
Assessment of participants’ on admission to study			
Assessment of treatment method under review			
Participants were stratified for the cervical cancer treatment method under review			
Ascertainment of cervical cancer and HIV status, prospectively from participants through diagnosis, laboratory tests and blood tests			
Ascertainment of cervical cancer and HIV status, retrospectively from participants through diagnosis, laboratory tests and blood tests			
Complete follow-up—all subjects accounted for			
Subjects lost to follow-up unlikely to introduce bias (≥ 75% follow-up or description provided of those lost			
If groups were not stratified for treatment methods and the distribution was unbalanced, were outcomes adjusted for

**Table 5:** Observational studies without a control group quality assessment checklist

Assessment criteria	Studies fulfilling criteria	Studies not fulling criteria
Study population was a consecutive cohort of participants		
Included participants have fulfilled predefined criteria		
Study design information given.

**Table 6:** Outcome measures’ quality assessment checklist

Cervical cancer treatment methods	Assessment criteria	Studies fulfilling criteria	Studies not fulling criteria
Surgery	Clinical definition		
	Technical investigation		
	Definition of treatment results		
Radiation therapy	Clinical definition		
	Technical investigation		
	Definition of treatment results		
Chemotherapy	Clinical definition		
	Technical investigation		
	Definition of treatment results		
Targeted therapy	Clinical definition		
	Technical investigation		
	Definition of treatment results		

Two reviewers among WF, TC and SF, will examine the quality and relevance of the extracted data by scoring each study from zero to five and through discussions; disagreements and discrepancies will be resolved [15].

### Data management

Literature search results will be saved in their respective database user accounts and citation records will be uploaded to EndNote Software, a reference package which facilitates the management of references and bibliographies.

### Synthesis

Results of this review will be synthesised using both the narrative synthesis and meta-analysis as described in the planned review [15]. Narrative synthesis will be used for descriptive analysis whilst random-effects aggregate data meta-analysis will be used to combine all numerical findings from the included studies. Assessment of bias will also be analysed using the meta-analysis and funnel plots will be produced using the RevMan software and statistical significance at 95% using *t* test will be inferred. Higgins and Thompson’s *I*^2^ statistic is going to be used to assess heterogeneity, where a 0% will indicate no heterogeneity and increase in percentage will indicate increase in heterogeneity, which will be significant at *p* value of less than or equal to 0.05 [17]. STATA Statistical package is going to be used to run the meta-analysis and tables and forest plots will be used to present estimates.

### Reporting

The PRISMA statement will guide this systematic review and its findings [12].

## Discussion

A systematic review of the published literature to identify different treatment methods that are currently being used to treat cervical cancer in HIV-seropositive women in developing countries will be undertaken. The review will investigate the different treatment methods of cervical cancer for HIV-seropositive women in developing countries and sources of heterogeneity within the studies. The evidence generated from this review will be used to address the gap that exist in this area as well as provide a basis for future research, cervical cancer policies and cervical cancer interventions. The strengths and limitations for this review will be considered and the review findings will be discussed in the context of other reviews and evidence that are relevant.

## Additional files

Additional file 1: Full-text screening. http://www.editorialmanager.com/sysr/download.aspx?id=31667&guid=f7b8a094-9955-42b6-b552-a4ecc0e9e636&scheme=1. (DOCX 15 kb)

Additional file 2: Data extraction form. http://www.editorialmanager.com/sysr/download.aspx?id=31668&guid=aa68746c-70e8-4763-b85a-c0db7bac60fa&scheme=1. (DOCX 18 kb)

Additional file 3: Newcastle-Ottawa Quality Assessment Scale. http://www.editorialmanager.com/sysr/download.aspx?id=31669&guid=0f0320bd-602b-4216-a6c6-bc7796072259&scheme=1. (DOCX 21 kb)

## Abbreviation

CINAHL: Cumulative Index to Nursing and Allied Health Literature
EMBASE: Excerpta Medica Database
HIV: Human immunodeficiency virus
HPV: Human papilloma virus
PRISMA-P: Preferred Reporting Items for Systematic Review and Meta-Analysis Protocols
RCT: Randomised controlled trial
WHO ICTRP: World Health Organization International Clinical Trials Registry Platform

## References

[1] Wu ES, Jeronimo J, Feldman S. Barriers and challenges to treatment alternatives for early-stage cervical cancer in lower-resource settings. Journal of Global Oncology. 0(0):JGO.2016.007369. 10.1200/JGO.2016.007369 PMC5646895 29094097

[2] World Health Organisation. Human papillomavirus (HPV). Available at http://www.who.int/immunization/topics/hpv/en/ [Accessed on 23 November 2015].

[3] Chirenje ZM (2005). HIV and cancer of the cervix. Best Pract Res Clin Obstet Gynaecol.

[4] Clifford GM, Polesel J, Rickenbach M. Cancer risk in the Swiss HIV cohort study: associations with immunodeficiency, smoking, and highly active antiretroviral therapy. J Natl Cancer Inst. 2005;97(6):425–32. 10.1093/jnci/dji072 15770006

[5] Moodley JR, Hoffman M, Carrara H, Allan BR, Cooper DD, Rosenberg L (2006). HIV and pre-neoplastic and neoplastic lesions of the cervix in South Africa: a case-control study. BMC Cancer.

[6] Finocchario-Kessler S, Wexler C, Maloba M, Mabachi N, Ndikum-Moffor F, Bukusi E (2016). Cervical cancer prevention and treatment research in Africa: a systematic review from a public health perspective. BMC Womens Health.

[7] Sherris J, Herdman C, Elias C (2001). Cervical cancer in the developing world. West J Med.

[8] Fletcher FE, Vidrine DJ, Tami-Meaury I, Danysh HE, King RM, Buchberg M, Arduino RC, Gritz ER (2014). Cervical cancer screening adherence among HIV-positive female smokers from a comprehensive HIV clinic. AIDS Behav.

[9] Viviano M, De Beaudrap P, Tebeu PM, Fouogue JT, Vassilakos P, Petignat P (2017). A review of screening strategies for cervical cancer in human immunodeficiency virus-positive women in sub-Saharan Africa. Int J Womens Health.

[10] Nakisigea C, Schwartz M, Ndira AO (2017). Cervical cancer screening and treatment in Uganda. Gynecologic Oncology Reports.

[11] Shamseer L. Moher D., Clarke M. Ghersi D. Liberati A. Petticrew M. Shekelle P. Stewart L.A. the PRISMA-P Group. Preferred Reporting Items for Systematic Review and Meta-Analysis Protocols (PRISMA-P) 2015: elaboration and explanation. Systematic Reviews. 2015;4:1 10.1186/2046-4053-4-1 PMC4320440 25554246

[12] Moher D, Liberati A, Tetzlaff J, Altman DG, The PRISMA Group. Preferred Reporting Items for Systematic Reviews and Meta-Analyses: the PRISMA statement. PLoS Med. 2009;6(7):e1000097. 10.1371/journal.pmed.1000097. 10.1371/journal.pmed.1000097 PMC2707599 19621072

[13] PROSPERO. http://www.crd.york.ac.uk/Prospero/. Accessed 15 Apr 2017.

[14] United Nations. Country classification. World Economic Situation and Prospects. 2012. http://www.un.org/en/development/desa/policy/wesp/wesp_current/2012country_class.pdf. Accessed 21 Apr 2017.

[15] Mapanga W, Elhakeem A, Feresu SA, Maseko F, Chipato T. Prevention of cervical cancer in HIV-seropositive women from developing countries: a systematic review protocol. Systematic Reviews Journal. 2017;6(91) 10.1186/s13643-017-0484-9. 10.1186/s13643-017-0484-9 PMC5404686 28438187

[16] The Newcastle-Ottawa Quality Assessment Scale. Available at http://www.ohri.ca/programs/clinical_epidemiology/nosgen.pdf.

[17] Higgins JP, Thompson SG (2002). Quantifying heterogeneity in a meta-analysis. Stat Med.

