# Supplementary material for: Treatment of cervical cancer in HIV-seropositive women from developing countries: a protocol for a systematic review
Source: Syst Rev. 2018 Jan 25;7:22. doi: 10.1186/s13643-018-0686-9 (PMC5785816; doi:10.1186/s13643-018-0686-9)
Supplement: Supplementary file 2 — Data extraction form. http://www.editorialmanager.com/sysr/download.aspx?id=31668&guid=aa68746c-70e8-4763-b85a-c0db7bac60fa&scheme=1. (DOCX 18 kb) [file 13643_2018_686_MOESM2_ESM.docx]

**Additional File 3**

Form 2 Data extraction: **TREATMENT OF CERVICAL CANCER IN HIV SEROPOSITIVE WOMEN FROM DEVELOPING COUNTRIES: A SYSTEMATIC REVIEW**

| *A. Reference details* | | | | |  |  |  |  |  |  |  |  |  |  |  |  |  |  |  |  |  |  |
| --- | --- | --- | --- | --- | --- | --- | --- | --- | --- | --- | --- | --- | --- | --- | --- | --- | --- | --- | --- | --- | --- | --- |
| A1. Ref ID, 1^st^ author, title, publication year, | | | | | | | | | |  | | | | | | | | | | | | |
| A2. Assessor’s name and date of assessment | | | | | | | | | |  | | | | | | | | | | | | |
| *B. Study details* | | |  | | | | | | |  |  |  |  |  |  |  |  |  |  |  |  |  |
| B1. Name of study/cohort | | | | | | | | | |  | | | | | | | | | | | | |
| B2. Design | | | | | | | | | | RCT | |  | Prospective cohort | | |  | | Other | | | |  |
| B2A. If other: | | | | | | | | | |  | | | | | | | | | | | | |
| B3. Country, setting | | | | | | | | | |  | | | | | | | | | | | | |
| *C. Cervical cancer treatment modalities* | | | | | | | | | | |  |  |  |  |  |  |  |  |  |  |  |  |
| C2. Type of treatment | | | | | | | | | | Surgery | |  | Chemotherapy | |  | Radiation Therapy | | |  | | Other |  |
| C2A. If other please describe | | | | | | | | | |  | | | | | | | | | | | | |
| C3. How ascertained | | | | | | | | | | Self-reported | |  | Objective methods | | | | | | | | |  |
| C4. Age ascertained | | | | | | | | | |  | | | | | | | | | | | | |
| C5A. If other: | | | | | | | | | |  | | | | | | | | | | | | |
| *D. Available participant numbers* | | | | | | | | |  |  |  |  |  |  |  |  |  |  |  |  |  |  |
| D1. Baseline | | | | | | | | | | Yes | |  | No | |  | If yes, number | | | | | |  |
| D2. Excluded | | | | | | | | | | Yes | |  | No | |  | If yes, number | | | | | |  |
| D3. Lost to follow-up | | | | | | | | | | Yes | |  | No | |  | If yes, number | | | | | |  |
| D4. Included in analysis | | | | | | | | | | Yes | |  | No | |  | If yes, number | | | | | |  |
| D5. All accounted for? | | | | | | | | | | Yes | |  | No | | | | | | | | |  |
| *E. Analysis* |  | | | | | | | | |  |  |  |  |  |  |  |  |  |  |  |  |  |
| E1. How results analysed | | | | | | | | | | Descriptive/ Trend | |  | Logistic regression | |  | Linear regression | | | |  | Other |  |
| E1A. If other: | | | | | | | | | |  | | | | | | | | | | | | |
| E2. Included in analysis | | | | | | | | | | Women | |  |  | |  | |  | | | | |  |
| E3. Only significant results presented? | | | | | | | | | | Yes | |  | No | | | | | | | | |  |
| *F. Summary of results* | | | | | |  | | | | | |  | | | | | | | | | | |
| F1. Prevalence/Mean difference | | | | | | | | | | Yes | |  | No | | | | | | | | |  |
| F2. Odds/Risk ratios | | | | | | | | | | Yes | |  | No | | | | | | | | |  |
| F3. Regression coefficients | | | | | | | | | | Yes | |  | No | | | | | | | | |  |
| F4. Confidence intervals (CIs)/ P-value/standard errors (SE) | | | | | | | | | | Yes | |  | No | | | | | | | | |  |
| F5. Other | | | | | | | | | | Yes | |  | No | | | | | | | | |  |
| F5A. If other | | | | | | | | | |  | | | | | | | | | | | | |
| *G. References for screening* | | | | | | |  | | | | |  |  |  |  |  |  |  |  |  |  |  |
| G1. Reference numbers | | | | | | | | | |  | | | | | | | | | | | | |
| *H. Effect estimates* | | | |  | | | | | |  |  |  |  |  |  |  |  |  |  |  |  |  |
| Association tested | | Number analysed | | | | | | Type of effect estimate and category comparison/value of unit change | | | | | | Effect estimate | | | 95% CI; SE; p-value | | | Confounders included in analysis | | |
| 1. | |  | | | | | |  | | | | | |  | | |  | | |  | | |
| 2. | |  | | | | | |  | | | | | |  | | |  | | |  | | |
