# Supplementary material for: Treatment of cervical cancer in HIV-seropositive women from developing countries: a protocol for a systematic review
Source: Syst Rev. 2018 Jan 25;7:22. doi: 10.1186/s13643-018-0686-9 (PMC5785816; doi:10.1186/s13643-018-0686-9)
Supplement: Supplementary file 3 — Newcastle-Ottawa Quality Assessment Scale. http://www.editorialmanager.com/sysr/download.aspx?id=31669&guid=0f0320bd-602b-4216-a6c6-bc7796072259&scheme=1. (DOCX 21 kb) [file 13643_2018_686_MOESM3_ESM.docx]

**Additional File 3**

Amended Newcastle-Ottawa Quality Assessment Scale: Treatment of cervical cancer in HIV seropositive women from developing countries: A systematic review.

Note: A study can be awarded a maximum of two stars for each numbered item – (except number 2 and 3 under case-control studies and number 5 under cohort studies).

CASE-CONTROL STUDIES

Selection

1) Is the case definition adequate?

a) Yes, with independent validation **

b) Yes, e.g record linkage or based on self-reports*

c) No description

2) Representativeness of the cases

a) Consecutive or obviously representative series of cases **

b) Potential for selection biases or not stated

3) Selection of Controls

a) Community controls **

b) Hospital controls *

c) No description

4) Definition of Controls

a) No history of disease (endpoint) *

b) No description of source

Comparability

1) Comparability of cases and controls on the basis of the design or analysis

a) Study controls for cervical cancer treatment modality (age, HIV status.) * *

b) Study controls for any additional factor * (parity, socio-economic status.)

Exposure

1) Ascertainment of exposure

a) Secure record (surgical/medical records, pathological/laboratory records) **

b) Structured interview where blind to case/control status *

c) Interview not blinded to case/control status

d) Written self-report or medical record only

e) No description

2) Same method of ascertainment for cases and controls

a) Yes *

b) No

3) Non-Response rate

a) Same rate for both groups *

b) Non respondents described

c) Rate different and no designation

COHORT STUDIES

Selection

1) Representativeness of the exposed cohort

a) Truly representative of the source population. **

b) Somewhat representative of the source population. *

c) Selected group of users e.g. nurses, volunteers.

d) No description of the derivation of the cohort.

2) Ascertainment of cervical cancer and HIV status

a) Prospectively from participants through diagnosis, laboratory tests and blood tests. **

b) Retrospectively collected with attempts to reduce recall bias (e.g. medical records and structured interview techniques). *

c) Retrospectively collected without attempts to reduce recall bias.

d) No description.

Comparability

3) Comparability of cohorts on the basis of the design/analysis

a) Study controls for HIV seropositive cervical cancer women. **

b) Study controls for any additional relevant factors (e.g. age, other diseases). *

c) Only unadjusted results presented.

Outcome

4) Assessment of prevention and treatment modality

a) Objective methods (prognosis, morbidity or mortality rates). **

b) Self-reported using validated questionnaire/diary/interview. *

c) Self-report.

d) No description.

5) Adequacy of cohort follow-up

a) Complete follow up - all subjects accounted for. *

b) Subjects lost to follow up unlikely to introduce bias (≥75% follow-up or description provided of those lost). *

c) <75% follow-up and no description of those lost.

d) No statement.

**RANDOMISED CLINICAL TRIALS**

**Selection**

1) Representativeness of the exposed group

a) Truly representative of the source population. **

b) Somewhat representative of the source population. *

c) Selected group of users e.g. patients, volunteers.

d) No description of the derivation of the group.

2) Ascertainment of cervical cancer and HIV status

a) Prospectively from participants through diagnosis, laboratory tests and blood tests. **

b) Retrospectively collected with attempts to reduce recall bias (e.g. medical records and structured interview techniques). *

c) Retrospectively collected without attempts to reduce recall bias.

d) No description.

**Comparability**

3) Comparability of groups on the basis of the design/analysis

a) Study controls for HIV seropositive cervical cancer women. **

b) Study controls for any additional relevant factors (e.g. age, other diseases). *

c) Only unadjusted results presented.

**Outcome**

4) Assessment of prevention modality

a) Objective methods (prognosis, morbidity or mortality rates). **

b) Self-reported using validated questionnaire/diary/interview. *

c) Self-report.

d) No description.

5) Adequacy of trial follow-up

a) Complete follow up - all subjects accounted for. *

b) Subjects lost to follow up unlikely to introduce bias (≥75% follow-up or description provided of those lost). *

c) <75% follow-up and no description of those lost.

d) No statement.

***Table 3: Randomised clinical trials quality assessment checklist***

| **Assessment criteria** | **Studies fulfilling criteria** | **Studies not fulling criteria** |
| --- | --- | --- |
| Randomization of participants is reported |  |  |
| All participants who entered the study would have been accounted for in the analysis |  |  |
| Participants were analysed in the groups they were randomized to |  |  |
| Blinded outcome assessment was used |  |  |
| Power calculation information was provided |  |  |
| Baseline characteristics of study groups were balanced or adjustment for the imbalance in analyses |  |  |

***Table 4: Observational studies with a control group quality assessment checklist***

| **Assessment criteria** | **Studies fulfilling criteria** | **Studies not fulling criteria** | **Studies not applicable** |
| --- | --- | --- | --- |
| Assessment of participants’ on admission to study |  |  |  |
| Assessment of treatment method under review |  |  |  |
| Participants were stratified for the cervical cancer treatment method under review |  |  |  |
| Ascertainment of cervical cancer and HIV status, prospectively from participants through diagnosis, laboratory tests and blood tests |  |  |  |
| Ascertainment of cervical cancer and HIV status, retrospectively from participants through diagnosis, laboratory tests and blood tests |  |  |  |
| Complete follow up - all subjects accounted for |  |  |  |
| Subjects lost to follow up unlikely to introduce bias (≥75% follow-up or description provided of those lost |  |  |  |
| If groups were not stratified for treatment methods and the distribution was unbalanced, were outcomes adjusted for |  |  |  |

***Table 5: Observational studies without a control group quality assessment checklist***

| **Assessment criteria** | **Studies fulfilling criteria** | **Studies not fulling criteria** |
| --- | --- | --- |
| Study population was a consecutive cohort of participants |  |  |
| Included participants have fulfilled predefined criteria |  |  |
| Study design information given. |  |  |

***Table 6: Outcome measures’ quality assessment checklist***

| **Cervical cancer treatment methods** | **Assessment criteria** | **Studies fulfilling criteria** | **Studies not fulling criteria** |
| --- | --- | --- | --- |
| Surgery | Clinical definition |  |  |
|  | Technical investigation |  |  |
|  | Definition of treatment results |  |  |
| Radiation therapy | Clinical definition |  |  |
|  | Technical investigation |  |  |
|  | Definition of treatment results |  |  |
| Chemotherapy | Clinical definition |  |  |
|  | Technical investigation |  |  |
|  | Definition of treatment results |  |  |
| Targeted therapy | Clinical definition |  |  |
|  | Technical investigation |  |  |
|  | Definition of treatment results |  |  |
